# Supplementary material for: Enhancing diversity analysis by repeatedly rarefying next generation sequencing data describing microbial communities
Source: Sci Rep. 2021 Nov 16;11:22302. doi: 10.1038/s41598-021-01636-1 (PMC8595385; doi:10.1038/s41598-021-01636-1)
Supplement: Supplementary file 1 — Supplementary Information. [file 41598_2021_1636_MOESM1_ESM.docx]

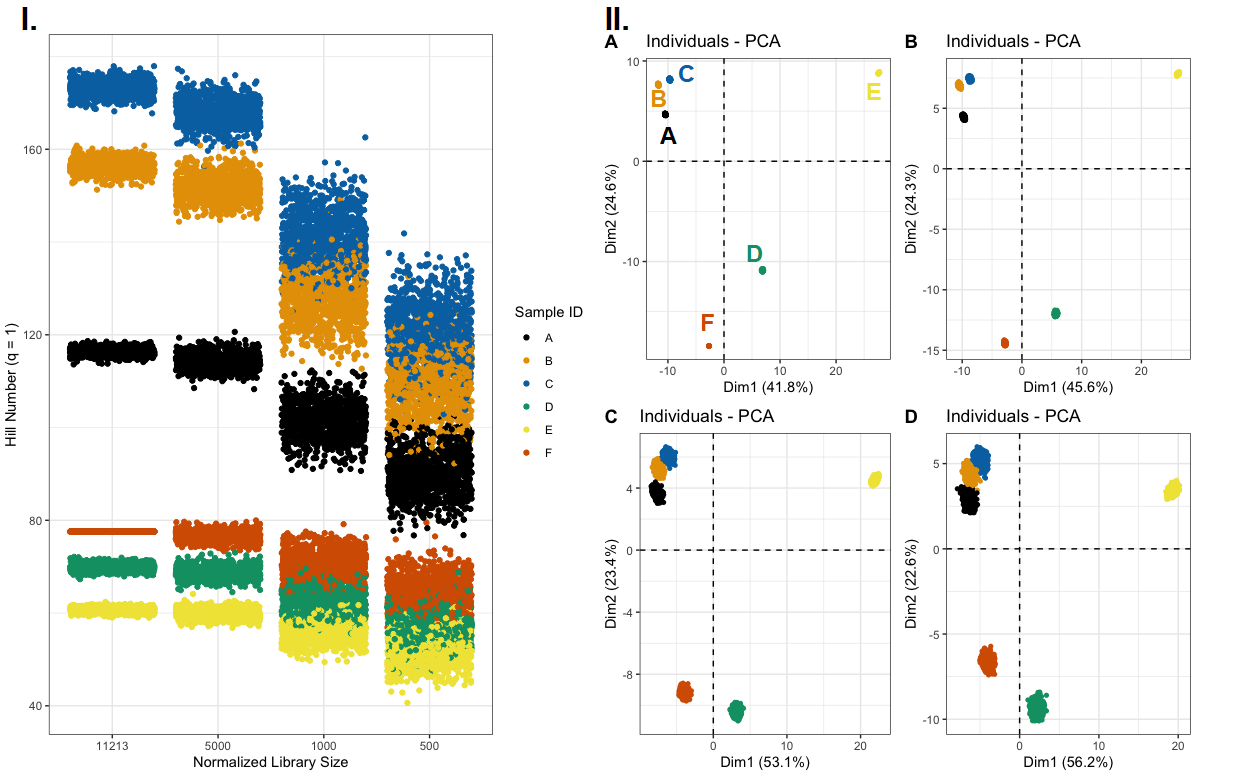


**Figure S1 : Evaluation of the impact of repeatedly rarefying on additional diversity metrics : the Hill Number (I) and the Jaccard distance (II). Six microbial communities were repeatedly rarefied to specified library sizes as indicated of 11,213 (II-A), 5000(II-B), 1000 (II-C) and 500 (II-D) .**


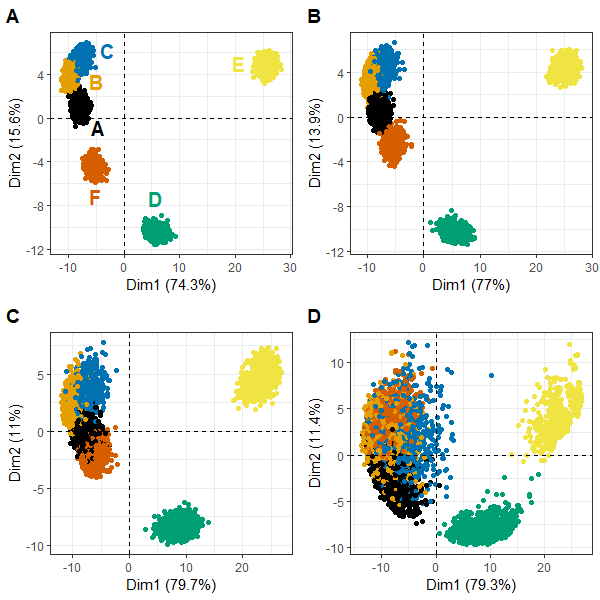


**Figure S2 Variation in PCA ordinations (using the Bray-Curtis dissimilarity on Hellinger transformed rarefied microbial communities) of six microbial communities repeatedly rarefied to very small library sizes of (A) 400, (B) 300, (C) 200 and (D) 100 sequences.**

Table S.1 Functions from other R packages used in mirlyn. *mirlyn* is an R package developed for library normalization and diversity analyses of amplicon sequencing and is available at [www.github.com/escamero/mirlyn](http://www.github.com/escamero/mirlyn).

| *mirlyn* function | Description | Functions used from other packages | Citations |
| --- | --- | --- | --- |
| bartax() | Generate taxonomic composition barcharts from taxonomic abundance data. | microbiome::transform(): generate compositional data from abundance data. | Leo Lahti et al.  microbiome R package.  URL: <http://microbiome.github.io> |
|  |  | phyloseq::tax_glom(): combine compositional data by desired taxonomic level.  ggplot2::ggplot(): plotting engine for all visualization. | Paul J. McMurdie and Susan Holmes (2013). phyloseq: An R package for reproducible interactive analysis and graphics of microbiome census data. PLoS ONE 8(4):e61217. |
| alphawhichDF() | Calculate alpha diversity values from sequence count data. | vegan::diversity(): used for alpha diversity calculation. | Jari Oksanen, F. Guillaume Blanchet, Michael Friendly, Roeland Kindt, Pierre Legendre, Dan McGlinn, Peter R. Minchin, R. B. O'Hara, Gavin L.    Simpson, Peter Solymos, M. Henry H. Stevens, Eduard Szoecs and Helene Wagner (2019). vegan: Community Ecology Package. R package version 2.5-6.    https://CRAN.R-project.org/package=vegan |

| Table S.1 Continued | | | |
| --- | --- | --- | --- |
| *mirlyn* function | Description | Functions used from other packages | Citations |
| alphacone() | Calculate alpha diversity values at different increments of library rarefaction for sequence data. | phyloseq::rarefy_even_depth(): rarefy taxonomic abundance data from multiple samples to an equal depth. | Paul J. McMurdie and Susan Holmes (2013). phyloseq: An R package for reproducible interactive analysis and graphics of microbiome census data. PLoS ONE 8(4):e61217. |
|  |  | vegan::diversity(): used for alpha diversity calculation. | Jari Oksanen, F. Guillaume Blanchet, Michael Friendly, Roeland Kindt, Pierre Legendre, Dan McGlinn, Peter R. Minchin, R. B. O'Hara, Gavin L.    Simpson, Peter Solymos, M. Henry H. Stevens, Eduard Szoecs and Helene Wagner (2019). vegan: Community Ecology Package. R package version 2.5-6.  <https://CRAN.R-project.org/package=vegan> |
| betamatPCA()  betamatPCA() | Principle component analysis of beta diversity values calculated from sequence count data. | vegan::vegdist(): calculate dissimilarity indices from taxonomic abundance data. | Jari Oksanen, F. Guillaume Blanchet, Michael Friendly, Roeland Kindt, Pierre Legendre, Dan McGlinn, Peter R. Minchin, R. B. O'Hara, Gavin L.    Simpson, Peter Solymos, M. Henry H. Stevens, Eduard Szoecs and Helene Wagner (2019). vegan: Community Ecology Package. R package version 2.5-6.  <https://CRAN.R-project.org/package=vegan> |
|  |  | vegan::decostand(): apply desired standardization to taxonomic abundance data prior to calculation of dissimilarity indices. |  |
|  |  | stats::prcomp(): principle component analysis. | R Core Team (2020). R: A language and environment for statistical computing. R Foundation for Statistical Computing, Vienna, Austria. URL  <https://www.R-project.org/> |
| mirl() | Repeated rarefaction of sequence count data. | phyloseq::rarefy_even_depth(): rarefy taxonomic abundance data from multiple samples to an equal depth. | Paul J. McMurdie and Susan Holmes (2013). phyloseq: An R package for reproducible interactive analysis and graphics of microbiome census data. PLoS ONE 8(4):e61217. |
| rarefy_whole_rep() | Repeated rarefaction of taxonomic abundance data at incremental library sizes. | phyloseq::rarefy_even_depth(): rarefy taxonomic abundance data from multiple samples to an equal depth. | Paul J. McMurdie and Susan Holmes (2013). phyloseq: An R package for reproducible interactive analysis and graphics of microbiome census data. PLoS ONE 8(4):e61217. |
| rarecurve() | Visualize observed ASV count from rarefy_whole_rep() output. | ggplot2::ggplot(): plotting engine for all visualization. | H. Wickham. ggplot2: Elegant Graphics for Data Analysis. Springer-Verlag New York, 2016. |
| alphawhichVis() | Visualize alpha diversity results from alphawhichDF(). | ggplot2::ggplot(): plotting engine for all visualization. | H. Wickham. ggplot2: Elegant Graphics for Data Analysis. Springer-Verlag New York, 2016. |
| betamatPCAvis()  betamatPCAvis() | Plot principle component analysis from betamatPCA(). | ggplot2::ggplot(): plotting engine for all visualization. | H. Wickham. ggplot2: Elegant Graphics for Data Analysis. Springer-Verlag New York, 2016. |
|  |  | factoextra::fviz_pca_ind(): wrapper for ggplot2 visualization of PCA data. | Alboukadel Kassambara and Fabian Mundt (2020). factoextra: Extract and Visualize the Results of Multivariate Data Analyses. R package version    1.0.7. <https://CRAN.R-project.org/package=factoextra> |
| phyloseqtodf() | Creation of a dataframe from a *phyloseq* object including taxonomy, ASV read counts and metadata. | tidyr::gather(): gathers columns for data frame reorganization. | Hadley Wickham and Lionel Henry (2020). tidyr: Tidy Messy Data. R package version 1.1.0. <https://CRAN.R-project.org/package=tidyr> |
| get_asv_table() | Generates a compiled ASV table with counts and taxonomic classification of individual amplicon sequence variants. | dplyr::mutate_if(): apply transformation to variables  dplyr::distinct(): subsets unique rows from dataframe. | Hadley Wickham, Romain François, Lionel Henry and Kirill Müller (2020). dplyr: A Grammar of Data Manipulation. R package version 1.0.0.  <https://CRAN.R-project.org/package=dplyr> |
| randomseqsig() | Identification of whether a taxonomic group is significantly dominant in the community using data shuffling. | reshape2::melt(): generates molten dataframe. | Hadley Wickham (2007). Reshaping Data with the reshape Package. Journal of Statistical Software, 21(12), 1-20. URL:  <http://www.jstatsoft.org/v21/i12/>. |
| plot_heat() | Generates heat maps visualizing the relative abundance of a taxonomic group of interest from a dataframe. | ggplot2::ggplot(): plotting engine for all visualization. | H. Wickham. ggplot2: Elegant Graphics for Data Analysis. Springer-Verlag New York, 2016. |
| asv_rename() | Assigns unique ASV I.D.’s to sequence variants. | readr::write_tsv(): generates tab delimited file from data frame object. | Hadley Wickham and Jim Hester (2020). readr: Read Rectangular Text Data. R package version 1.4.0. <https://CRAN.R-project.org/package=readr> |
| fasta_rename() | Assigns corresponding unique ASV identifiers to a FASTA file. | Biostrings::readDNAStringSet(): reads FASTA format file.  Biostrings::writeXStringSet(): generates FASTA file.  readr::read_tsv(): reads tab delimited file into data frame. | H. Pagès, P. Aboyoun, R. Gentleman and S. DebRoy (2020). Biostrings: Efficient manipulation of biological strings. R package version 2.58.0.  <https://bioconductor.org/packages/Biostrings> |
| fullbartax() | Generates taxonomic composition bar charts for specified taxonomic levels. | ggplot2::ggplot(): plotting engine for all visualization. | H. Wickham. ggplot2: Elegant Graphics for Data Analysis. Springer-Verlag New York, 2016. |
